# Editorial: Serum metabolites in diagnostics and therapeutics

**DOI:** 10.3389/fmolb.2024.1528799

**Published:** 2024-11-28

**Authors:** Gregorio Peron, Donghai Lin

**Affiliations:** ^1^ Department of Molecular and Translational Medicine, University of Brescia, Brescia, Italy; ^2^ Department of Chemical Biology, College of Chemistry and Chemical Engineering, Xiamen University, Xiamen, China

**Keywords:** serum, metabolites, pathogens, disease treatment, serum metabolome, molecular, mechanisms

The field of serum metabolomics is revolutionizing our understanding of disease mechanisms, and at the same time offers unprecedented opportunities in diagnostics and personalized medicine. This Research Topic entitled “*Serum Metabolites in Diagnostics and Therapeutics*” presents a collection of research articles that, overall, show the potential of metabolomics to capture real-time biochemical snapshots: especially this aspect makes metabolomics a valuable approach for early disease detection, therapeutic monitoring, and biomarker discovery. By examining the latest methodologies and their clinical applications, this Research Topic is intended as an integrated perspective on the advancements and clinical applications of serum metabolomics.

Serum metabolomics, which assesses small-molecule metabolites circulating in blood, directly reflects metabolic processes affected by disease, lifestyle, and genetic factors. Metabolites like amino acids, lipids, and nucleotides often reveal distinctive patterns in disease states, allowing clinicians to observe shifts linked to cellular dysfunction and pathological pathways (Qiu et al., 2023). Recent studies highlight the value of these metabolic signatures in early detection, as even minor changes in metabolite levels can indicate disease onset before clinical symptoms emerge (Al-Sulaiti et al., 2023). For instance, the literature emphasizes that metabolomics has wider applications in clinics than genomic and proteomic approaches because it provides a dynamic readout of the current physiological state (Ramautar et al., 2013). In fact, unlike genomics that reflects potential risk, metabolomics presents a functional snapshot, making it ideal for real-time monitoring.

The improvement of analytical technologies such as ultra-performance liquid chromatography (UHPLC) and high-resolution mass spectrometry (MS) has played a pivotal role in the progress of serum metabolomics. These techniques allow to efficiently separate the chemical constituents of a complex matrix like serum and detect metabolites at trace levels, being this crucial for profiling minor but diagnostically significant changes of serum metabolic composition. For instance, in a study published in this Research Topic, UHPLC-MS/MS was used to identify complex lipid profiles linked to cardiovascular and inflammatory diseases, providing new insights into vascular health. High-throughput nuclear magnetic resonance (NMR) spectroscopy is also widely used to perform serum metabolomics and especially for metabolite identification. Because it allows for robust and non-destructive analysis of samples, it is suitable for routine clinical applications. Together, UHPLC-MS and NMR allow for comprehensive metabolomic profiling and can highlight biomarkers that could serve as early indicators for conditions like cancer, diabetes, and neurodegenerative diseases.

Computational advancements in machine learning (ML) and artificial intelligence (AI) have also catalyzed the field’s growth. The interpretation of high-dimensional data generated by metabolomics is often challenging and requires sophisticated tools to identify patterns and relationships between metabolites and disease phenotypes (Rattray et al., 2018). Several studies in this Research Topic integrated AI and multivariate statistics to enhance predictive capabilities: for instance, deep learning models have been used to classify patients based on serum profiles and predict the outcomes of metabolic syndrome. This AI-driven approach enhances predictive accuracy by modeling complex, nonlinear relationships that cannot be managed with traditional statistical methods. Moreover, as metabolomic data grows, ML techniques can adaptively improve, increasing their potential applications (e.g., cancer detection and monitoring of chronic diseases).

The clinical applications of serum metabolomics extend across diagnostics, disease classification, and therapeutic management. In cancer, early detection remains crucial for improving patient outcomes (Brockhoven et al., 2023), and the analysis of serum biomarkers can represent a non-invasive and accessible alternative to biopsies. For instance, in a study of this Research Topic authors identified lipid biomarkers that can be potentially monitored for distinguishing malignant states. Also, serum metabolites may have a role as indicators of therapeutic efficacy of cancer therapies, as highlighted in another article, particularly in chemotherapy response. Furthermore, the possibility to track metabolic changes over time allows clinicians to adjust therapies based on patient-specific responses, promoting more personalized treatments that reduce adverse effects.

Similar considerations can be made for chronic diseases like diabetes and cardiovascular disease. A study published in this Research Topic explored metabolic profiles that predict treatment outcomes in patients with type 2 diabetes and identified serum markers linked to insulin resistance and glucose regulation. This highlights metabolomics as a tool not only for diagnosis but also for tracking longitudinal metabolic changes, supporting timely intervention. Similarly, in inflammatory diseases, metabolomics can detect shifts in lipid and amino acid profiles that correlate with inflammatory markers, offering insights into disease mechanisms and potential intervention points.

Despite these advancements, challenges remain in translating serum metabolomics from research into clinical practice, particularly in terms of standardization and reproducibility. The variability in metabolite measurement across different platforms and protocols can complicate the validation of biomarkers. As one article in the Research Topic advocates, harmonizing analytical protocols—standardizing sample preparation, instrument calibration, and data processing—is essential for achieving reproducible and comparable results. This standardization would not only facilitate clinical adoption but also support large-scale biomarker validation studies, which are essential for establishing diagnostic thresholds and reference ranges in metabolomics.

Ultimately, the research presented in this Research Topic, coupled with recent findings from wider literature, highlights serum metabolomics as a cornerstone of precision diagnostics. By capturing dynamic biochemical snapshots, serum metabolomics not only allows for early disease detection but also supports personalized medicine, enabling treatment decisions that align with the patient’s unique metabolic profile. The integration of AI with metabolomics, combined with the continued refinement of analytical technologies and standardization efforts, promises a future where metabolomics becomes routine in medical diagnostics and therapeutic monitoring. Continued investment in these areas is essential to unlock the full potential of serum metabolomics, transforming it from a powerful research tool into a staple of clinical practice.

To conclude, as the Guest Editors, we would like to thank all the Authors that contributed to this Research Topic by publishing their research, as well as the Reviewers and the Assistant and Academic Editors for their valuable support.
